# Development of Amorphous AlN Thin Films on ITO-Glass and ITO-PET at Low Temperatures by RF Sputtering

**DOI:** 10.3390/mi16090993

**Published:** 2025-08-29

**Authors:** Miriam Cadenas, Michael Sun, Susana Fernández, Sirona Valdueza-Felip, Ana M. Diez-Pascual, Fernando B. Naranjo

**Affiliations:** 1 Universidad de Alcalá, Grupo de Ingeniería Fotónica, Unidad Asociada Instituto de Óptica-CSIC, Tecnologías Fotónicas y de Sensores, 28871 Alcalá de Henares, Spain; michael.sun@uah.es (M.S.); sirona.valdueza@uah.es (S.V.-F.); fernando.naranjo@uah.es (F.B.N.); 2 Departamento de Energía, CIEMAT, Av. Complutense 40, 28040 Madrid, Spain; susanamaria.fernandez@ciemat.es; 3 Universidad de Alcalá, Facultad de Ciencias, Departamento de Química Física e Ingeniería Química, 28871 Alcalá de Henares, Spain; am.diez@uah.es

**Keywords:** AlN thin films, reactive sputtering, flexible electronics, optoelectronic devices

## Abstract

Aluminum nitride (AlN) is a material of wide interest in the optoelectronics and high-power electronics industry. The deposition of AlN thin films at elevated temperatures is a well-established process, but its implementation on flexible substrates with conductive oxides, such as ITO-glass or ITO-PET, poses challenges due to the thermal degradation of these materials. In this work, the deposition and characterization of AlN thin films by reactive sputtering at a low temperature (RT and 100 °C) on ITO-glass and ITO-PET substrates are presented. The structural, optical, and electrical properties of the samples have been analysed as a function of the sputtering power and the deposition temperature. XRD analysis revealed the absence of peaks of crystalline AlN, indicative of the formation of an amorphous phase. EDX measurements performed on the ITO-glass substrate with a radiofrequency power applied to the Al target of 175 W confirmed the presence of Al and N, corroborating the deposition of AlN. SEM analyses showed the formation of homogeneous and compact layers, and transmission optical measurements revealed a bandgap of around 5.82 eV, depending on the deposition conditions. Electrical resistivity measurements indicated an insulating character. Overall, these findings confirm the potential of amorphous AlN for applications in flexible optoelectronic devices.

## 1. Introduction

Among semiconductor materials, aluminum nitride (AlN) is a material of wide interest in the optoelectronics and high-power electronics industry due to its high thermal stability, high thermal conductivity (single crystal: 285 W/m·K), and excellent electrical insulation (ρ ~10^11^–10^13^ Ω cm). Its wide band gap (~6.2 eV in its crystalline phase) also makes it an attractive material for applications in optical windows in the deep UV, sensors, light-emitting diodes, laser diodes, and high-frequency transistors [1,2]. Costly bulk AlN substrates boost interest in the growth of wide band-gap semiconductors on cheap silicon (Si), sapphire (Al_2_O_3_), or silicon carbide (SiC) substrates [3,4,5]. Typically, III-N layers can be grown on Si substrates or on flexible substrates with conductive oxides, such as ITO-glass or ITO-PET, by metal–organic chemical vapor deposition (MOCVD) [6] or molecular-beam epitaxy (MBE) [7]. However, these methods need high temperatures to attain high-quality films, which result in mismatches in the lattice with the formation of abundant dislocations or even cracks in the AlN film [7,8]. Therefore, alternative approaches are required to control the deposition of AlN films at relatively low temperatures that preserve the optical and electrical properties of AlN without compromising the integrity of the substrate [9,10].

The sputtering technique is an inexpensive technology that allows the deposition of AlN crystalline layers on numerous substrates (sapphire, silicon, glass, plastics, etc.) and a wide range of temperatures (from RT to above 800 °C) [11]. In the process of sputtering, a plasma is created between a cathode or target material to be sputtered and the anode, usually the substrate holder or chamber. An inert gas is used as a sputtering gas to avoid unintentional chemical reactivity [12]. The energy source for sustaining the plasma can be DC, RF, or a pulsed DC power supply. DC-magnetron sputtering is a cost-effective process, but several concerns arise during reactive sputtering for obtaining insulating thin films from metallic targets, such as defects in the thin film and reduced lifetime of targets caused by arcing (high current and low voltage). The use of a radio frequency (rf) power source instead of a DC power source in reactive sputtering avoids the charge accumulation at the target surface and reduces arcing-related problems, thus leading to higher quality films. However, a number of parameters need to be optimized, including sputtering power, process gas pressure, substrate temperature, substrate to target distance, film thickness, etc. [13].

Despite numerous studies having attempted the deposition of AlN thin films by RF sputtering on Si-based substrates [14,15,16], articles on the deposition of flexible substrates such as ITO-glass or ITO-PET are very scarce. Deposition of layers on this type of substrate presents unique challenges, principally due to the inherent flexibility of the substrate and potential for deformation under stress. These challenges include film cracking, debonding, and changes in electrical properties under strain. Also, the lower processing temperatures required for flexible substrates limit the deposition techniques that can be used. Further, most studies have focused on the growth of crystalline AlN films, while the properties of amorphous AlN have hardly been investigated [17]. Crystalline AlN has a higher meting point, thermal conductivity, hardness, and better piezoelectric properties, while amorphous AlN has been reported to have better optical properties and higher transparency [18], hence is more suitable for transparent electronic applications and other uses, e.g., as passivation and dielectric layers, high-k gate oxides, corrosion resistance coatings, and advanced phase-change memory [17]. In this work, we describe for the first time the optimization of the deposition of amorphous AlN on ITO-glass or ITO-PET in reactive N_2_ atmosphere by reactive RF sputtering at RT and 100 °C, and its structural, optical, and electrical properties are studied. Specifically, the influence of the radiofrequency (rf) power applied to the Al target (P_Al_) and the growth temperature on the quality of the obtained films is analyzed, with the aim of evaluating their viability for applications in flexible electronic and advanced optoelectronic devices.

## 2. Materials and Methods

The samples under study were deposited on commercial ITO-glass and ITO-PET using a reactive RF sputtering system (AJA International, ATC ORION-3-HV). Before placing the substrates in the sputtering chamber, they were cleaned in organic solvents (10′ acetone, except for the ITO-PET substrate, and 10’ methanol) and dried with nitrogen. The target of 2-inch Al (5N purity) was pre-sputtered with Ar (6N purity) prior to the growth. Next, in the case of deposits at 100 °C, the substrates were degassed in the growth chamber at 100 °C for 30 min and cleaned with Ar plasma by applying an RF power of 10 W to the substrate under a chamber pressure of 10 mTorr. However, in the case of RT deposits, no substrate degassing was performed, only soft cleaning with Ar plasma. For the AlN growth, N_2_ (6N) was used as the reactive gas, with a flow rate of 14 sccm. The substrate–target distance and sputtering pressure were kept at 10.5 cm and 0.47 Pa, respectively. The deposition conditions were fixed, taking into account former studies of the research group [19,20].

The growth of AlN was performed on sapphire, ITO-glass, and ITO-PET, investigating the effect of the power applied to the aluminum target P_Al_ (100, 125, 150, and 175 W) on the quality of the AlN film.

The crystal structure, thickness, and density of the AlN layers were investigated by X-ray diffraction (XRD) analysis with an XPERT-PRO diffraction system, using Cu K_α1_ incident radiation (λ = 0.154 nm) at 45 kV and 40 mA. A high-resolution optical configuration was used, including a parabolic W/Si graded incident beam mirror and Soller slits (0.04 rad), combined with a parallel plate collimator (0.18°) on the diffracted beam side. The detection was carried out using a PIXcel1D RTMS detector in receiving slit mode. The angle ranged from 20° to 80°, with increments of 0.02° to study the crystal structure in Bragg–Brettano configuration, while X-ray reflectometry (XRR) measurements were carried out to estimate the density and thickness of the AlN samples. For the latter case, measurements were performed in the reflection mode with a 2θ-Ω geometry, using a step size of 0.01° over a range from 0.1° to 8.0°.

The surface roughness of the AlN layers was measured by atomic force microscopy (AFM) in the tapping mode with a Bruker NanoScope V multimode microscope at 20 °C in a liquid environment. Measurements were acquired using rectangular silicon cantilevers with a nominal spring constant of 42 N/m and a resonance frequency of 320 kHz. The nominal value of the tip’s radius of curvature was 2 nm, and the scanning speed during the acquisitions ranged from 10 to 400 nm/s. Visualization and data processing were conducted using Nanoscope Analysis 2.0 software.

The cross-section and surface morphology of the layers were assessed with a JEOL JSM-7600F field emission scanning electron microscope (FESEM). Micrographs were acquired in COMPO mode, at an accelerating voltage of 15.0 kV, a working distance of 8.0 mm, and a magnification of 45,000×. The scale bar in the images corresponds to 100 nm. Measurements were performed under high vacuum conditions to ensure optimal resolution.

Optical transmission measurements were performed at RT and normal incidence using a Perkin Elmer Lambda 1050 UV–VIS–NIR spectrophotometer (PerkinElmer, Waltham, MA, USA).

Electrical AlN layer resistivity was estimated using the transfer length method (TLM) with 50 × 100 um aluminum contacts deposited by thermal evaporation with contact spacing of (10, 30, 60, 90, and 120 um) and an SMU 2450 KEITHLEY device to register the corresponding I–V curves [21].

## 3. Results and Discussion

### 3.1. Structural Characterization

**Figure 1:**
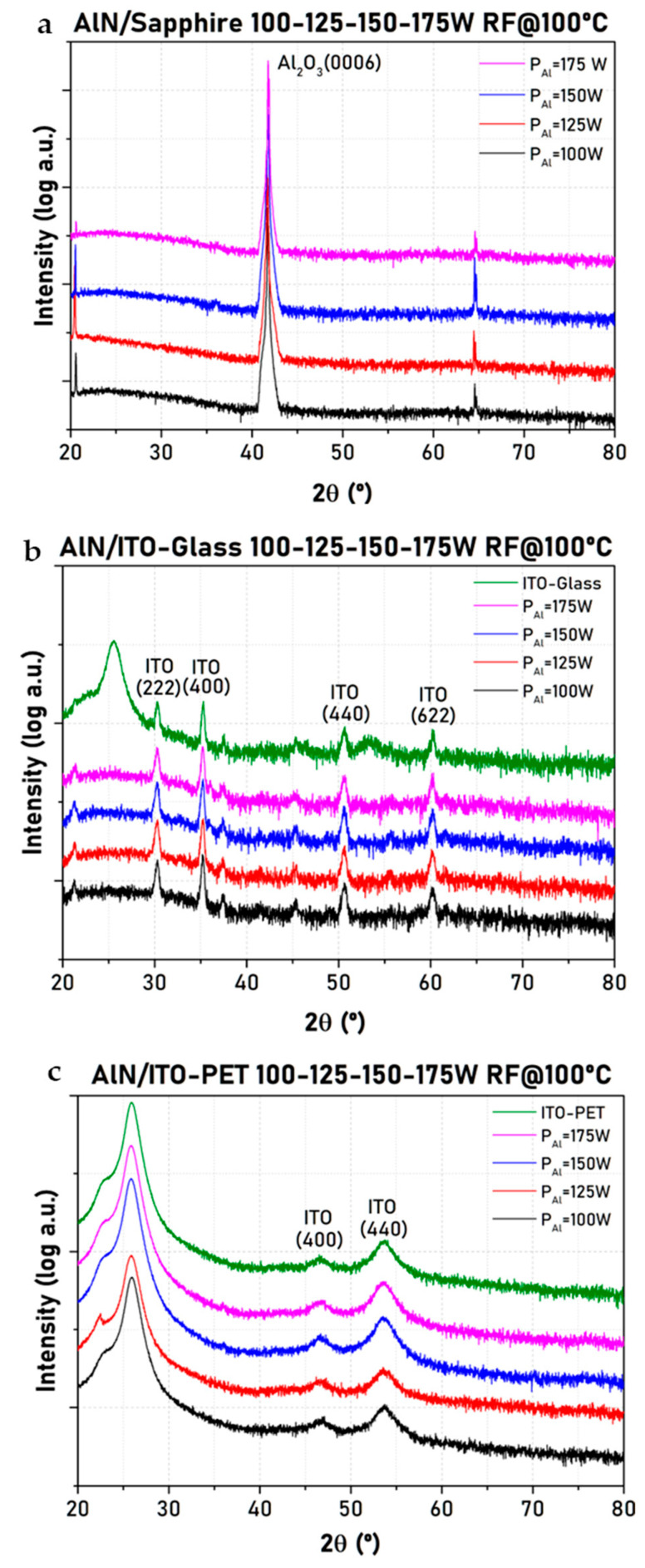
X-ray diffractograms of AlN films deposited on sapphire (**a**), ITO-glass (**b**), and ITO-PET (**c**) at 100 °C and different P_Al_.

The properties of AlN films grown on ITO-glass and ITO-PET have been studied as a function of the power applied to the aluminum target (P_Al_). The X-ray diffractograms of films deposited on sapphire in the same run were also measured as reference, and ITO-glass and ITO-PET at 100 °C are shown in Figure 1. Very similar results were obtained for samples deposited at RT. XRD analysis revealed the absence of characteristic peaks of crystalline AlN for any of the P_Al_ employed (expected around 2θ ~36° for the (0002) plane [11]) in the three samples, pointing out the possible formation of an amorphous phase. This is in contrast to the results found for AlN films deposited on Si substrates at 450 °C, which showed a decrease in the intensity of the (0002) crystalline peak of AlN upon decreasing P_Al_ [11]. Thus, the crystallinity of AlN films deposited via sputtering is known to depend on the substrate temperature. Highly crystalline films are obtained by heating the substrate (250–500 °C) [22], while at lower temperatures, the AlN films could exhibit an amorphous state. Even at 100 °C, amorphous AlN does not crystallize because the temperature is not high enough to provide the necessary energy for atoms to overcome energy barriers and rearrange into a crystalline structure. This behavior could be expected considering the high melting point of the AlN material, 2000 °C [23], which is related to the high bond energy between Al and N. It should be noted that in Figure 1, some minor peaks have not been indexed because they do not correspond to peaks of interest in the substrate or the AlN layer.

**Figure 2:**
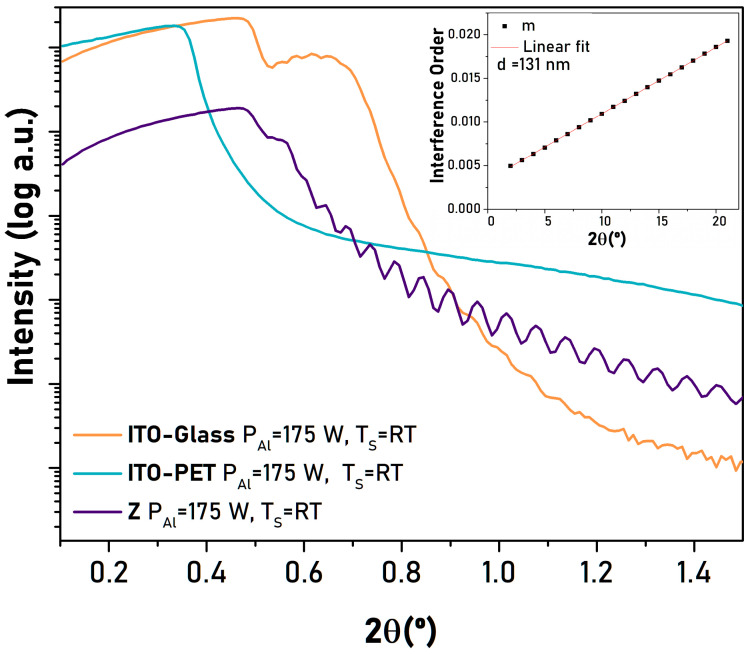
X-ray reflectivity curves for AlN films deposited on sapphire (purple), ITO-glass (orange), and ITO-PET (green) at RT and a P_Al_ of 175 W. The inset shows the fitting to obtain the thickness for the film grown on sapphire (see explanation in the text).

Figure 2 shows the X-ray reflectivity curves (reflectivity intensity vs. 2θ) obtained for the AlN film deposited on sapphire, ITO-glass, and ITO-PET at RT and with P_Al_ = 175 W. A logarithmic scale is used in the Y-axis due to the wide dynamic range of the reflectivity intensity. The reflectivity profile shows oscillations, named Kiessig fringes [24], caused by an interference between the X-rays reflected from the surface of the AlN film and the interface between the AlN film and the substrate. The oscillations depend on the film thickness and the difference between the densities of the film and its substrate, and the sharpness of the interface layer/substrate and layer surface/air [25]. The thicker the film, the shorter the period of the oscillations. The larger the density difference and interface sharpness, the higher the amplitude of the oscillation. Clear oscillations in the intensity are observed for the layer deposited on sapphire, while these oscillations are much smaller and almost absent in the layers grown on ITO-glass or ITO-PET, respectively, which could be related to a less abrupt AlN/ITO interface.

The X-ray reflectivity method was used to determine the film density and thickness [25] using the following equations:

(1) $$\frac{1}{d}=\frac{2\sqrt{{\sin\left(\theta \left(rad\right)\right)}^{2}-{\sin\left(\theta_{c}\left(rad\right)\right)}^{2}}}{m\lambda }$$

(2) $$\rho =\frac{2\delta \pi }{r_{e}N_{A}\lambda^{2}}\frac{\sum x_{i}A_{i}}{\sum x_{i}Z_{i}}$$

(3) $$\delta =\frac{{\theta_{c}}^{2}}{2}$$

where *d* is the film thickness in nm, *θ_c_* is the critical angle for total reflection, *λ* is the X-ray wavelength, m the interference order, *ρ* is the density in g/cm^3^, 1-δ is the real part of the refractive index (*n)* for X-ray radiation, *Z_i_* is the atomic number, *A_i_* is the molar mass, *x_i_* is the atomic ratio, N_A_ is the Avogadro number, and r_e_ is the radius of an electron (2.818 nm).

The inset in Figure 2 shows the fitting to obtain the film thickness for the AlN film grown on sapphire, in which the order of the interference is plotted against the angle at which it occurs, obtained from the XRR measurement. Table 1 summarizes the calculated thickness (*d*) values (Equation (1)) for the AlN films deposited at different temperatures and different P_Al_ powers. In the case of films grown on ITO-PET, it was not possible to estimate the thickness since the amplitude of the oscillations was too small, as discussed earlier. Thickness values range from 50 to 138 nm for target powers of 100 and 175 W, corresponding to growth rates of 25 and 65 nm/h, respectively.

Systematically, the film thickness increases with increasing target power, which is in agreement with previous studies on the deposition of thin films by sputtering [26,27]. The velocity of sputtered atoms increases with the RF power, and a higher speed results in a longer mean free path; hence, the sputtered atoms experience a lower number of collisions, resulting in a higher number of atoms arriving at the substrate, thus leading to a higher packing density and consequently, higher thickness. Further, the increase in target power for a given pressure results in an increase in the plasma density, and more Al atoms get sputtered, leading to an increase in the deposition rate.

Table 2 collects the calculated density values (Equation (2)) for the AlN films grown at different temperatures and target powers. Again, it was not possible to calculate the density for films grown on ITO-PET. The density obtained at RT is consistent with the theoretical value calculated for amorphous AlN (2.966 g/cm^3^) [28], about 10% lower than that of crystalline AlN due to the lack of long-range ordering.

No clear trend in the film density is found with increasing target power. A plausible explanation for the lack of correlation observed here could be that, even at low target powers, the substrate surface is quickly covered with a monolayer of Al atoms, meaning that the deposited film is already continuous. Consequently, further increases in power do not significantly affect the film density. The same lack of trend is observed with increasing substrate temperature. In general, for crystalline layers, the density increases with increasing substrate temperature due to a larger crystallite size [29]. However, the density of an amorphous film is expected to decrease with increasing substrate temperature, due to an increase in the porosity of the films [30]. Differences in density values for the films deposited on the two substrates are very small, pointing towards a similar nucleation and growth mechanism. Thus, the nuclei are expected to grow up independently and then coalesce until a continuous film is formed, following the typical processes of the Volmer–Weber growth mode [31]. When the complete surface is covered by the AlN film, the surface becomes smoother, in agreement with SEM and AFM measurements, as will be discussed later. Overall, results indicate that it is possible to attain controlled and precise control of the AlN growth on these substrates, hence it is ideal to be used as a buffer layer in solar cells.

### 3.2. Morphological Characterization

**Figure 3:**
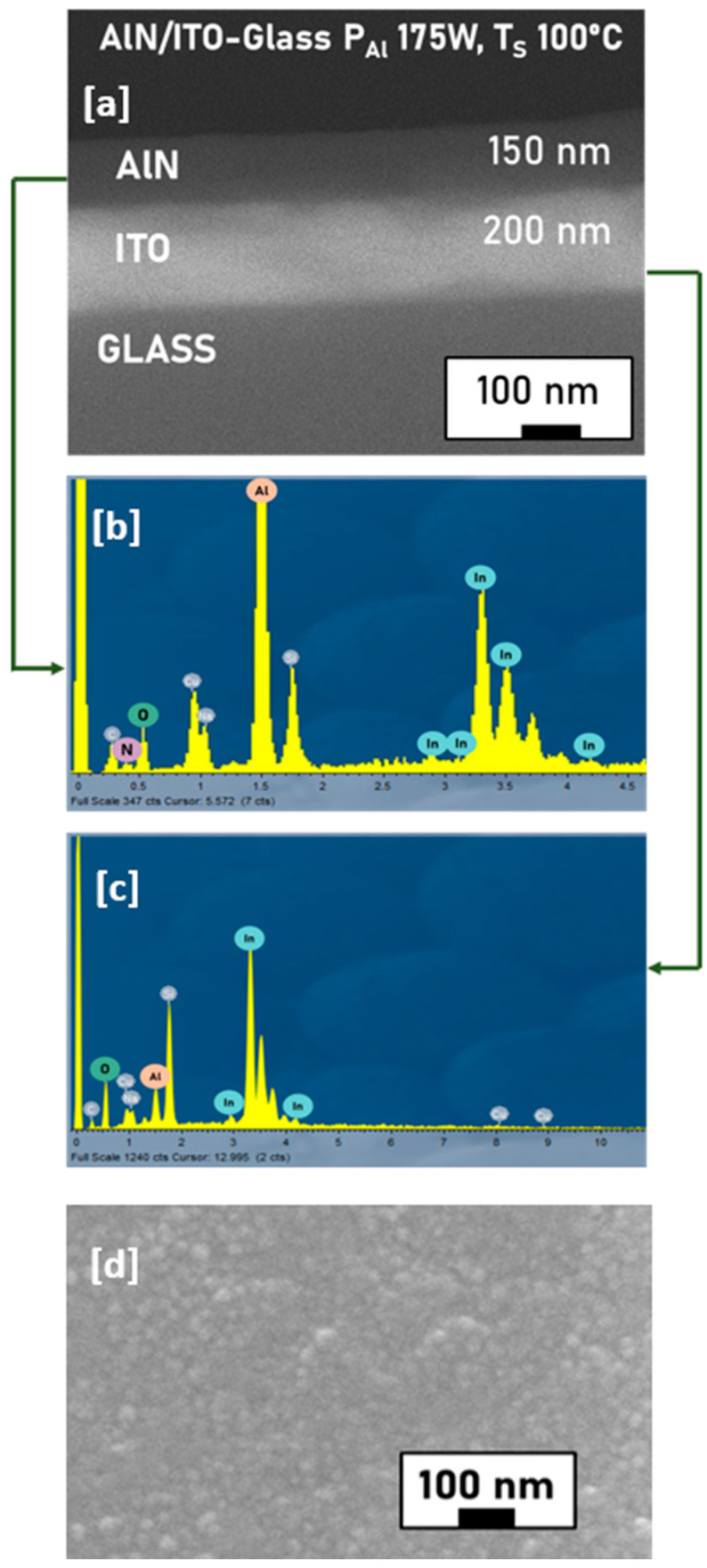
Cross-section and surface ((**a**,**d**), respectively) SEM images of the AlN layer on the ITO-glass substrate with P_Al_ = 175 W, deposited at 100 °C. EDX measurements on the AlN layer and the ITO layer of the same sample ((**b**,**c**), respectively).

The surface and cross-section morphology of the as-deposited thin films were investigated using SEM-EDX analysis. Representative micrographs and EDX spectra for AlN grown on ITO-glass at a power of 175 W and a substrate temperature of 100 °C are shown in Figure 3. Similar morphology was found for the samples prepared at different powers and temperatures. The surface and cross-sections were smooth, compact, uniform, crack-free, and featureless (Figure 3a,d), pointing out the low roughness of the thin films, in agreement with studies on other amorphous thin films [32]. It is also indicative of the complete coverage of the surface, as inferred from XRR spectra. Former studies [33] reported that the deposition of amorphous oxides via sputtering favored the atom-by-atom growth of the thin film, which resulted in a compact and uniform surface and cross-section morphology, similar to the present study.

**Figure 4:**
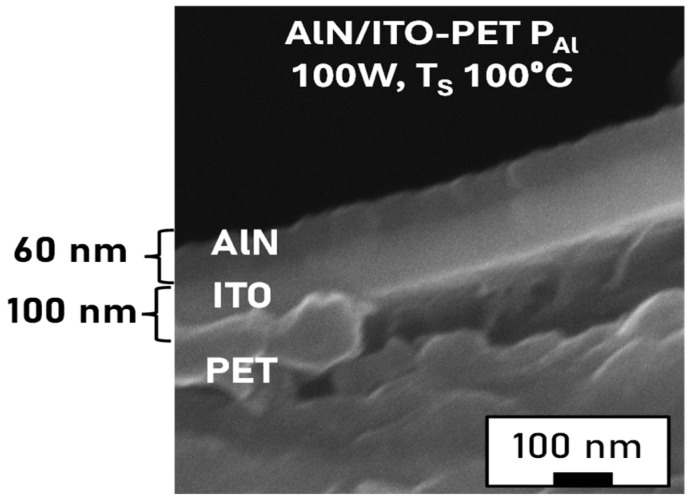
Cross-section SEM image of the AlN layer on the ITO-PET substrate with P_Al_ = 100 W, deposited at 100 °C.

The thickness of the AlN layer obtained from the cross-section SEM images was, in all cases, in good agreement with that found from XRR. Further, EDX spectra (Figure 3b) confirmed the presence of Al and N in the top layer, corroborating the successful growth of the film. The spectrum obtained is consistent with previous studies reported in the literature, which showed the EDX spectrum for an AlN film on a Si substrate prepared at a substrate temperature of 200 °C [34]. Theoretically, the atomic ratio of Al:N in the film should be 1:1, while the peak intensity of Al in the spectrum is much higher than that of N (Figure 3b). This can be explained considering that the atomic percentage of N cannot be quantified from EDX since the detection efficiency for low-Z atoms is very low [35]. Moreover, a significant fraction of the K_α_ X-rays coming from the N is absorbed by the beryllium window that separates the sample chamber from the analyzer. Additionally, since AlN is a poor electrical conductor, it will possibly be locally over-heated by the primary electrons, which, in turn, may easily cause a loss of volatile atoms like nitrogen. The EDX spectrum of ITO-glass reveals the presence of the characteristic peaks of In and O (Figure 3c), in agreement with previous studies [36]. Sn cannot be detected since its peaks overlap with those of In, and its atomic percentage is very low (3–4%). Similar conclusions were drawn from the SEM-EDX analysis of samples deposited on ITO-PET (Figure 4). The surface was characteristic of an amorphous film, with flat, compact, and dense layers, without individual particles. The cross-section images showed a lower film thickness compared to those grown on ITO-glass, which could be related to the lower root mean square (rms) roughness calculated from AFM images.

Despite the difficulty of determining thicknesses by XRR in ITO-PET due to its low reflectivity, cross-sectional images obtained by SEM (Figure 4) confirm the formation of continuous and compact layers.

**Figure 5:**
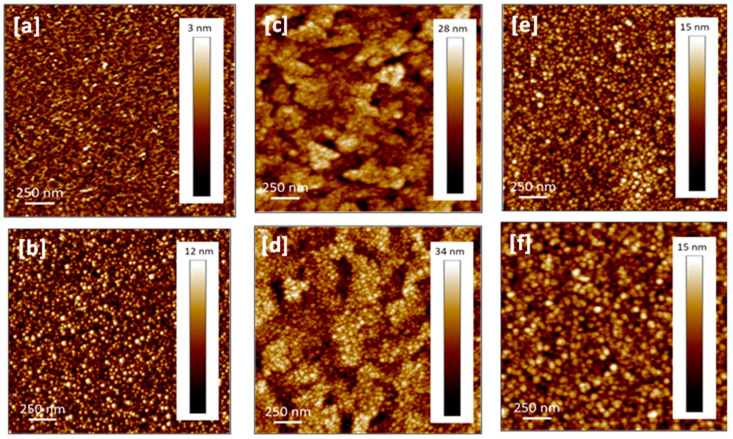
AFM images of AlN film grown on sapphire (**a**,**b**), ITO-glass (**c**,**d**), ITO-PET (**e**,**f**) at a power of 100 W (**a**,**c**,**e**) and 175 W (**b**,**d**,**f**), deposited at 100 °C.

**Figure 6:**
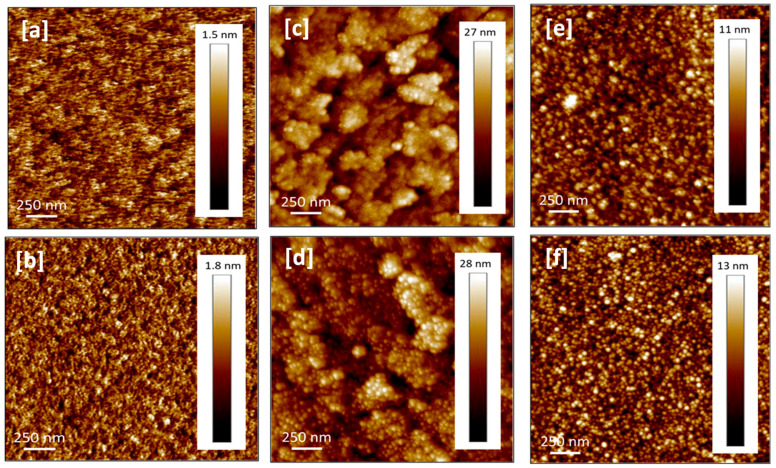
AFM images of AlN film grown on sapphire (**a**,**b**), ITO-glass (**c**,**d**), ITO-PET (**e**,**f**) at a power of 100 W (**a**,**c**,**e**) and 175 W (**b**,**d**,**f**), deposited at RT.

AFM analysis was carried out to obtain additional information about the surface morphology of the AlN film, and representative images of AlN/ITO-glass, AlN/ITO-PET, and AlN/sapphire grown at powers of 100 W and 175 W at 100 °C and RT, respectively, in an area of 2 × 2 µm^2^ are shown in Figure 5 and Figure 6, respectively. A very similar morphology was observed at both temperatures.

According to AFM images, the surface of the coatings is compact and continuous, without individual particles or conglomerates, in agreement with SEM images. The films show a relatively smooth surface, without voids or discontinuities, compatible with an amorphous nature, with rms roughness values in the range of 2–2.5, 4–5, and 2–0.5 nm at 100 °C for films grown on ITO-PET, ITO-glass, and sapphire, respectively, being in the range of 1.5–2, 4, and < 1 nm for samples deposited at RT, on the same substrates, respectively. These values can be seen more clearly in Table 3.

These values were calculated as the average of the roughness obtained in areas of 5 × 5, 2 × 2, and 1 × 1 µm^2^ for each case. These ranges given on each substrate were obtained from the average roughnesses of the four powers used in this study (100, 125, 150, and 175 W). It can be clearly observed that films on ITO-glass show higher roughness, which is consistent with the XRR spectra that exhibited a smaller oscillation amplitude for this sample. The roughness values obtained in this work are slightly higher than those reported for amorphous AlN deposited via RF sputtering on a Si substrate at high temperatures (about 1.4 nm at 450 °C) [37], while they are in good agreement with those found by Pigeat et al. [38] (about 4 nm for amorphous AlN deposited on Si substrate at 70 °C). Thus, it is confirmed again that the substrate temperature plays a key role in the surface morphology, and particularly, in the film roughness.

No clear trend in surface roughness change was found as a function of P_Al_ when growing on ITO-PET and ITO-glass; on the contrary, for samples grown on sapphire, a small increase in roughness appears with increasing power. However, it would be expected that increasing the power would produce particles with high energy at the target and therefore increase the bombardment of high kinetic energy particles on the substrate, resulting in higher surface roughness values [39], as observed on sapphire. In the case of ITO-PET and ITO-glass substrates, the surface roughness of the AlN samples deposited on top is highly affected by the initial surface roughness of the substrate (2.3 nm and 4.1 nm, for ITO-PET and ITO-glass substrates, respectively), hindering the emerging of the small dependence between surface roughness and Pal observed on sapphire, where the initial rms surface roughness of the substrate is much lower (below 1 nm).

It is worth noting that the low surface roughness of the AlN films on the ITO-PET substrate, as indicated by AFM (rms < 3 nm), supports their applicability in flexible electronics. Although this study did not include mechanical bending tests, the structural integrity and smoothness of the AlN films deposited on flexible ITO-PET substrates suggest good compatibility with flexible electronics.

### 3.3. Optical Characterization

**Figure 7:**
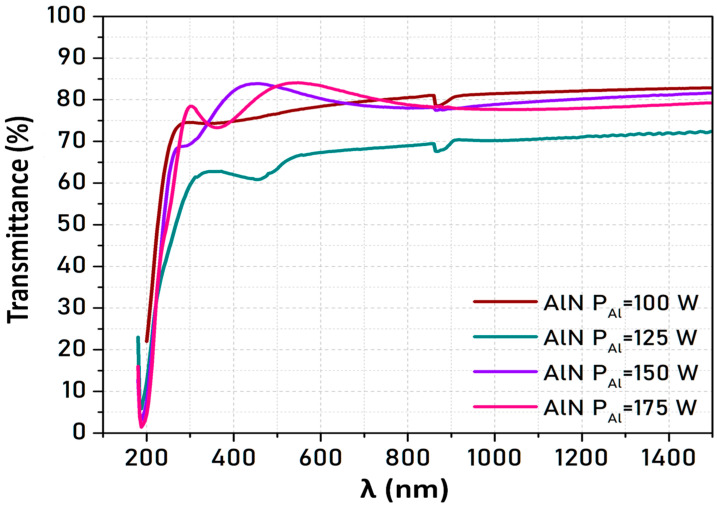
Percentage of transmittance as a function of the wavelength for AlN films deposited on sapphire at 100 °C and different P_Al_ values.

Figure 7 shows the transmittance spectra of AlN deposited on sapphire at 100 °C for different P_Al_ values. For all the films, the transmittance increases with increasing wavelength from 200 to 300 nm (corresponding to the absorption band-edge) and then remains almost constant (transparency region). In the case of AlN films deposited on ITO-glass and ITO-PET, it is not possible to observe the absorption band-edge of the AlN layer, as its energy is above the band gap energy of the ITO layer. The reduction in transmittance observed in the sample deposited at P_Al_ = 125 W can be attributed to a higher density of structural defects, which can enhance light scattering and absorption.

The optical band gap (Eg) was calculated from the transmittance spectra. Specifically, the method used to obtain the band gap energy from the transmittance measurements is described in detail in reference [40]. The results obtained for AlN films grown at different temperatures and P_Al_ on a sapphire substrate are compared in Table 4. It should be pointed out that in amorphous semiconductors, a band-tail arising from the crystallographic disorder interlopes in the absorption spectrum into the gap region, making the absorption edge difficult to determine experimentally.

The estimated value of E_g_ slightly increased with increasing P_Al_. This could be explained considering that increasing the energy of the Al atoms could reduce the number of defects in the films, which would lead to a larger bandgap [41].

All the values obtained in this study are below the theoretical value for crystalline AlN (6.2 eV). This may be related to the presence of defects and electronic states within the band gap in the disordered structure of the amorphous form, which, as mentioned above, leads to a reduction in the absorption edge of the band gap. Further, the results obtained are in very good agreement with those reported by other authors for amorphous AlN thin films deposited at low temperatures (i.e., 5.84 eV at T < 50 °C [42]).

### 3.4. Electrical Characterization

**Figure 8:**
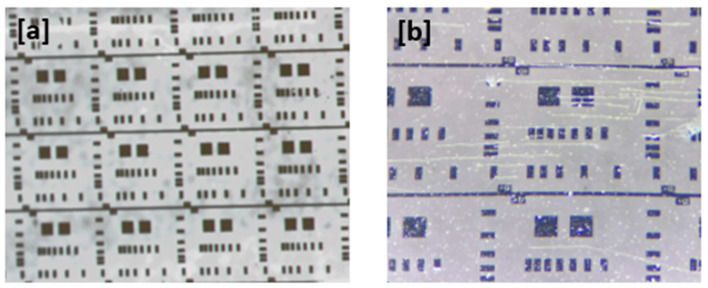
Images of the transfer length method test structure for the characterization of Al/AlN contact resistance: (**a**) array of rectangular Al contacts; (**b**) Al contacts deposited on ITO-PET. The square contacts measure 150 × 150 µm in both images.

The Transfer Length Method or “Transmission Line Model” (TLM) is a technique widely used in semiconductors to determine the specific contact resistivity between a metal and a semiconductor [21]. The goal is the determination of the specific contact resistivity of a metal–semiconductor junction. For such a purpose, an array of rectangular Al contacts (100 nm) was deposited on the surface of AlN, as is depicted in Figure 8.

In the TLM method, the total resistance R_T_ is given by the expression [21]:

R_T_ = 2R_c_ + (R_s_/Z) L (4)

where R_c_ is the contact resistance, R_s_ is the sheet resistance of the semiconductor substrate, Z is the width of the metal pads, and L is the contact spacing. The plot of R_T_ vs. L allows us to calculate R_s_, and the obtained values for AlN layers grown on ITO-glass and ITO-PET at different temperatures and powers are given in Table 5.

All the films grown on sapphire were found to be insulating. However, the resistivities of AlN grown on ITO-PET and ITO-glass were slightly lower. Parallel conduction was found in some samples, especially those deposited on ITO-PET, attributed to the presence of cracks. In addition to crack formation, local film irregularities or defects at the AlN/ITO interface could also contribute to the occasional conductivity observed in ITO-PET samples; confirming this would require additional characterization techniques, which will be addressed in future work. As seen in Table 5, this only occurs in samples deposited at 100 °C, while those deposited at RT are insulating. The values found in this work are consistent with those reported for bulk and surface AlN (in the range of 10^8^–10^9^ Ω cm [43]). Overall, this high electrical resistivity will be beneficial for applications in semiconductor devices, since it minimizes the risk of electrical leakage and shorts.

## 4. Conclusions

This study demonstrated the feasibility of depositing amorphous AlN thin films on ITO-glass and ITO-PET substrates at low temperatures using reactive sputtering. The influence of the power applied to the Al target (P_Al_) and the growth temperature on the quality of the obtained films has been analyzed. The resulting films exhibited a homogeneous, compact morphology and a smooth surface, without voids or discontinuities, compatible with an amorphous nature. RMS roughness values in the range of 1.5–3, 4–5, and < 1 nm were obtained from AFM images for films grown on ITO-PET, ITO-glass, and sapphire, respectively. According to XRR spectra, the film thickness increased with increasing target power and deposition temperature, ascribed to improved adhesion and packing density. A maximum transmittance of around 80% was attained for all the samples, independently of the film thickness. A band gap energy of around 5.82 eV has been calculated, below the theoretical value for crystalline AlN (6.0–6.2 eV). Further, results show a small increase in the band gap energy when increasing the power applied to the Al target. All the samples exhibited high electrical resistivity, in agreement with reported values for thin AlN layers. Overall, results indicate that amorphous AlN films deposited on ITO-glass and ITO-PET can be a viable alternative for applications in flexible electronics and optoelectronic devices such as flexible sensors, displays, actuators, batteries, supercapacitors, or transparent electrodes.

## Figures and Tables

**Table 1:** Thickness values (nm) for AlN films deposited at different temperatures and different P_Al_ powers on sapphire and ITO-glass substrates.

P_Al_ (W)	Sapphire 100 °C	Sapphire RT	ITO-Glass 100 °C	ITO-Glass RT
100	56	52	65	50
125	76	82	85	85
150	109	106	----	----
175	138	131	130	130

**Table 2:** Density values (g/cm^3^) for AlN films deposited at different temperatures and different P_Al_ powers on sapphire and ITO-glass substrates.

P_Al_ (W)	Sapphire 100 °C	Sapphire RT	ITO-Glass 100 °C	ITO-Glass RT
100	2.75	2.86	2.81	2.79
125	2.81	2.81	2.80	2.74
150	2.71	2.69	2.65	2.69
175	2.73	2.77	2.75	2.71

**Table 3:** Average RMS roughness values obtained by AFM for AlN films on sapphire, ITO-glass, and ITO-PET substrates at P_Al_ of 100 and 175 W, as well as at deposition temperatures of 100 °C and room temperature.

P_Al_ (W)	Sustrate	Means RMS (nm) at 100 °C	Means RMS (nm) at RT
100	Sapphire	0.5	0.3
	ITO-glass	4.1	4.1
	ITO-PET	2.1	1.6
175	Sapphire	2	0.3
	ITO-glass	4.9	3.9
	ITO-PET	2.3	2.0

**Table 4:** Band gap values (eV) calculated from the transmittance spectra for AlN films deposited at different temperatures and different P_Al_ powers on sapphire and ITO-glass substrates.

P_Al_ (W)	Sapphire 100 °C	Sapphire RT
100	5.47	5.83
125	5.77	5.77
150	5.76	5.58
175	5.82	5.93

**Table 5:** Sheet resistance (Ω/□) calculated using the TLM method for AlN films deposited at different temperatures and different P_Al_ powers on ITO-glass and ITO-PET substrates. Insulating refers to measurement data > 1 × 10^9^ Ω/□.

P_Al_ (W)	ITO-Glass 100 °C	ITO-Glass RT	ITO-PET 100 °C	ITO-PET RT
100	Insulating	Insulating	Insulating	Insulating
125	Insulating	1.30 × 10^7^	5.21 × 10^5^	Insulating
150	Insulating	Insulating	8.31 × 10^4^	Insulating
175	Insulating	Insulating	1.51 × 10^3^	Insulating

## Data Availability

The original contributions presented in this study are included in the article. Further inquiries can be directed to the corresponding authors.
